# Robotic pendant drop: containerless liquid for μs-resolved, AI-executable XPCS

**DOI:** 10.1038/s41377-023-01233-z

**Published:** 2023-08-18

**Authors:** Doga Yamac Ozgulbas, Don Jensen, Rory Butler, Rafael Vescovi, Ian T. Foster, Michael Irvin, Yasukazu Nakaye, Miaoqi Chu, Eric M. Dufresne, Soenke Seifert, Gyorgy Babnigg, Arvind Ramanathan, Qingteng Zhang

**Affiliations:** 1 Data Science and Learning Division, Argonne National Laboratory, Lemont, IL 60439 USA; 2 X-ray Science Division, Argonne National Laboratory, Lemont, IL 60439 USA; 3 Departement of Computer Science, University of Chicago, 5801 S Ellis Ave, Chicago, IL 60637 USA; 4 XRD Design and Engineering Department, Rigaku Corporation 3-9-12 Matsubara-cho, Akishima-shi, Tokyo 196-8666 Japan; 5 Bioscience Division, Argonne National Laboratory, Lemont, IL 60439 USA

**Keywords:** Lasers, LEDs and light sources, Electronics, photonics and device physics

## Abstract

The dynamics and structure of mixed phases in a complex fluid can significantly impact its material properties, such as viscoelasticity. Small-angle X-ray Photon Correlation Spectroscopy (SA-XPCS) can probe the spontaneous spatial fluctuations of the mixed phases under various in situ environments over wide spatiotemporal ranges (10^−6^–10^3^ s /10^−10^–10^−6^ m). Tailored material design, however, requires searching through a massive number of sample compositions and experimental parameters, which is beyond the bandwidth of the current coherent X-ray beamline. Using 3.7-μs-resolved XPCS synchronized with the clock frequency at the Advanced Photon Source, we demonstrated the consistency between the Brownian dynamics of ~100 nm diameter colloidal silica nanoparticles measured from an enclosed pendant drop and a sealed capillary. The electronic pipette can also be mounted on a robotic arm to access different stock solutions and create complex fluids with highly-repeatable and precisely controlled composition profiles. This closed-loop, AI-executable protocol is applicable to light scattering techniques regardless of the light wavelength and optical coherence, and is a first step towards high-throughput, autonomous material discovery.

## Abstract (web summary)

Robot-compatible liquid sample setup for a fully-automated, μs-resolved Small-angle X-ray Photon Correlation Spectroscopy (SA-XPCS) workflow that can enable autonomous designs of complex fluids.

## Graphical abstract

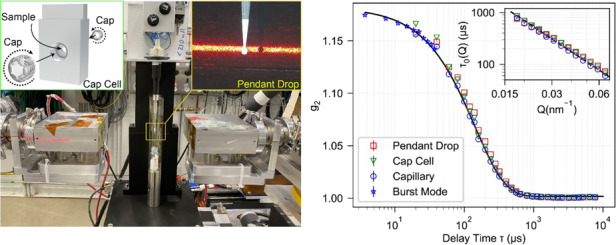

## Introduction

Complex fluid is a class of materials with a stable mixture of phases that is microscopically disordered but macroscopically homogeneous^1^. When certain conditions are met, complex fluids undergo phase separations, forming structures at mesoscopic length scales which are responsible for orders-of-magnitude changes in material properties, e.g., opacity^2,3^, diffusivity^4^, viscoelasticity^5^, and conductivity^6,7^. Precise control of the dynamic pathway of these mesoscopic structures can therefore lead to materials with tunable and even rewritable properties, offering exciting opportunities in numerous material science advances such as neuromorphic computing^8^ and targeted drug delivery^9^ that rely on programmable and switchable material properties with large on/off ratios.

Despite its scientific importance, the evolution of structural dynamics in phase-transitioning complex fluids is difficult to capture with scanning imaging methods (e.g., electron microscopy) due to its spatially hierarchical nature, i.e., the macroscopic material property change resulted from mesoscopic structure formation is driven by stochastic motions at a microscopic scale. Despite lacking the direct imaging capacity, time-resolved x-ray scattering provides in situ and real-time information on the evolution of spatial ordering over hierarchical scales (10^−10^–10^−6^ m) thanks to the use of hard x-ray beam and the recent development of high-speed pixel array detectors^10–12^. In addition, with the Free-Electron Laser and the “laser-like” coherent x-ray beam from synchrotron undulator sources^13^, X-ray photon correlation spectroscopy (XPCS), the expansion of dynamic light scattering (DLS) into x-ray wavelength, can directly probe the time scales of spontaneous fluctuation of spatial orderings via temporal decorrelation of scattered intensities. Enhanced by the sub-nm wavelength and strong penetration power of the hard x-ray beam, the wide range of spatiotemporal sensitivity of XPCS has led to unique insights in a myriad of material science systems, including collective motion of nanoparticles in a polymer matrix^14,15^, in-operando mesoscale mass flow during 3D printing^16^, and glass transition and gelation in dense colloids^17^.

One of the major challenges in XPCS studies of complex fluids is the unrealistically large amount of combinatorial sample conditions arising from a multi-dimensional parameter space (e.g., temperature, composition, synthesis history, etc.). Autonomous material discovery combining AI and automated experimental protocols have proven to be an effective method in navigating large parameter space and optimizing material design^18,19^. However, despite the high level of automation at macromolecular crystallography synchrotron X-ray beamlines^20–26^, the scope of hardware automation at coherent X-ray scattering beamlines (i.e., XPCS, X-ray coherent diffraction imaging, X-ray ptychography) is quite limited in general, primarily because the throughput of coherent x-ray scattering measurements has been traditionally limited by the amount of available coherent x-ray flux. This limitation is now being gradually lifted by the worldwide construction and commissioning of fourth-generation x-ray sources^27–30^, which increases the coherent flux by at least 10^2^ and the measurement throughput by 10^4^, leading to a reduction of sample turnaround time from as long as 15 h^31^ to less than 5 s. Development of high-throughput, closed-loop XPCS protocol is therefore opportune and can be further combined with AI-assisted XPCS analysis^32,33^ to achieve self-driving, autonomous design of complex fluids with tailored material properties.

Containerless liquid, e.g., acoustic levitation^34^ and pendant drop^35^, has shown potentials for automated sample delivery at synchrotron x-ray beamlines. However, XPCS on containerless liquid has not been demonstrated, primarily because of the potential drifting and convection flow issues that can contaminate XPCS results acquired using a micro-focused X-ray beam. In addition, XPCS on complex fluid with high fluidity, such as suspensions of sub-μm nanoparticle, micelles or viruses in aqueous solvents, has not been possible until recently with the development of the μs-resolved single-photon-counting pixel array detector. In this work, we demonstrate that XPCS measurements on ~100 nm diameter silica nanoparticles suspended in a drop of water (i.e., pendant drop) are consistent with those from a generic liquid sample container (e.g., a sealed quartz capillary), therefore validating the use of pendant drop as a sample container for light scattering studies of complex fluids. The pendant drop is generated using an electronic pipette, and can be aspirated back into the pipette tip after the measurement for automated sample disposal. We also demonstrate that the electronic pipette can be mounted on a robotic arm for automated liquid handling, i.e., accessing various stock solutions and creating samples with new composition profiles. This leads to a precisely controlled, highly-repeatable, and AI-executable liquid XPCS experimental protocol. This protocol can also be easily incorporated into other light scattering techniques beyond synchrotron x-ray community, such as DLS and Small-Angle X-ray Scattering (SAXS) at rotating-anode x-ray source, providing common ground for multimodal, collaborative characterization on complex fluids and paving the path to autonomous discoveries of soft materials.

## Results

### Validation of pendant drop for XPCS

**Fig. 1:**
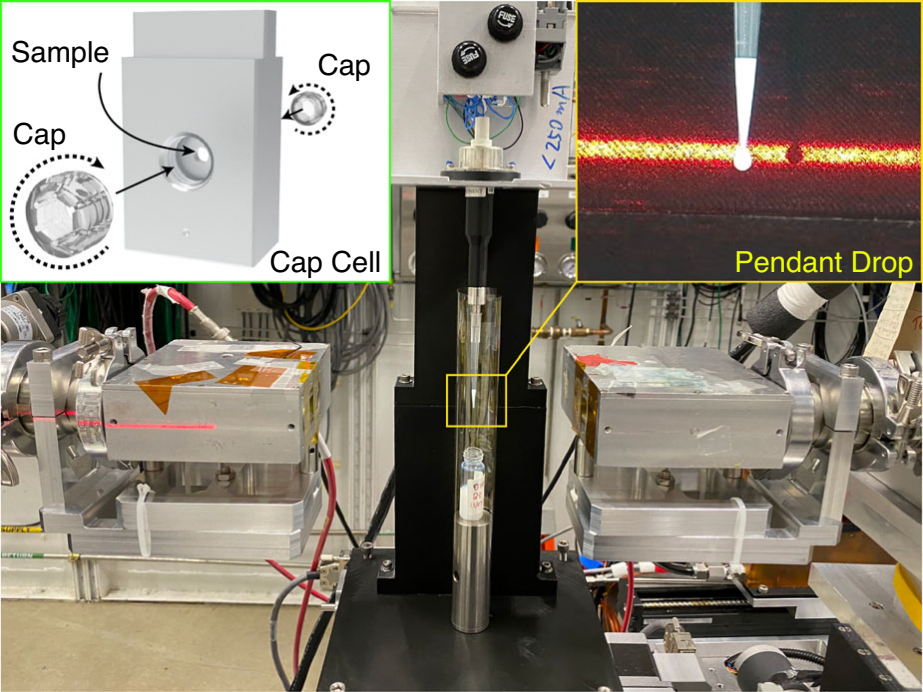
SA-XPCS at APS Beamline 8-ID-I. The sample is a dilute suspension of ~100 nm diameter silica nanoparticles in water (2.5% volume fraction). The top-left inset shows the 3D mechanical drawing of the aluminum liquid cell with externally threaded polycarbonate caps, and the top-right inset shows a close-up image of the pendant drop. The pendant drop is generated using an electronic pipette and can be aspirated back into the pipette tip at the end of the measurement. The red laser beam marks the height of the X-ray beam for coarse vertical alignment. A 0.13-mm-thick polycarbonate sleeve is placed around the pendant drop to suppress airflow and solvent evaporation

**Fig. 2:**
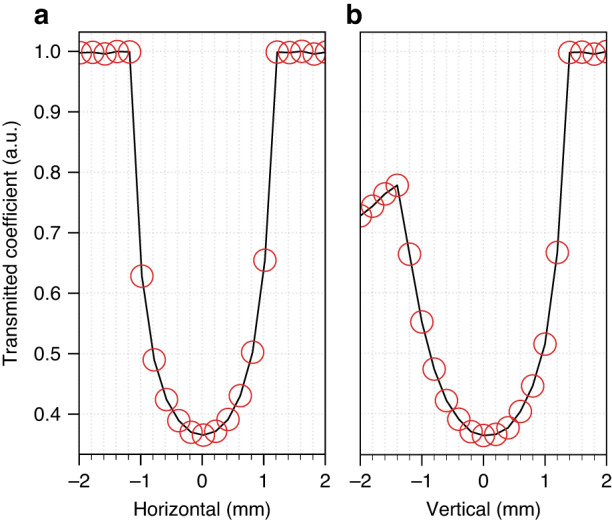
Spatial alignment of the pendant drop with the x-ray beam. The shape and position of the pendant drop is indicated by the x-ray transmission coefficient as a function of the **a** horizontal and **b** vertical sample position. The beam is aligned with the center of the pendant drop for the SAXS and XPCS measurements in Figs. 3–5

Small-angle XPCS (SA-XPCS) combining SAXS and XPCS measurements were performed at Beamline 8-ID-I of the Advanced Photon Source (APS), Argonne National Laboratory. The sample is ~100 nm silica nanoparticles suspended in water with a volume fraction of 2.5%. This colloidal system can be approximated as hard spheres and is sufficiently dilute so that the nanoparticle dynamics follow Einstein–Stokes diffusion. The pendant drop setup is shown in Fig. 1. The drop was generated using an electronic pipette and was illuminated by a 10 μm × 10 μm coherent x-ray beam at 10.94 keV photon energy with an energy spread of 0.03% and a flux of 1.3 × 10^10^ photons-per-second. Details regarding the beamline and the x-ray optics can be found in previous studies^36^. The center of the pendant drop is found by monitoring the x-ray transmission coefficient as a function of the in-plane sample stage translation perpendicular to the x-ray beam (Fig. 2). The pendant drop remains stable during the stage translation and the horizontal alignment scan shows a pendant drop diameter of 2.4 mm. The pendant drop is surrounded by a 0.13 mm thick polycarbonate sheet to reduce the airflow and suppress the solvent evaporation, whose effect on XPCS results will be discussed in more detail in Fig. 5. In order to validate the use of pendant drop setup for SA-XPCS measurements, the SAXS and XPCS results from the silica nanoparticle sample in the pendant drop are compared with reference results from two different types of containers, i.e., a sealed thin-walled quartz capillary and a miniature cell assembly designed for viscous and solid samples (Cap Cell, Fig. 1). Technical details regarding the capillary and the Cap Cell setup can be found in Figs. S1–S4 and the Method and Materials Section.

**Fig. 3:**
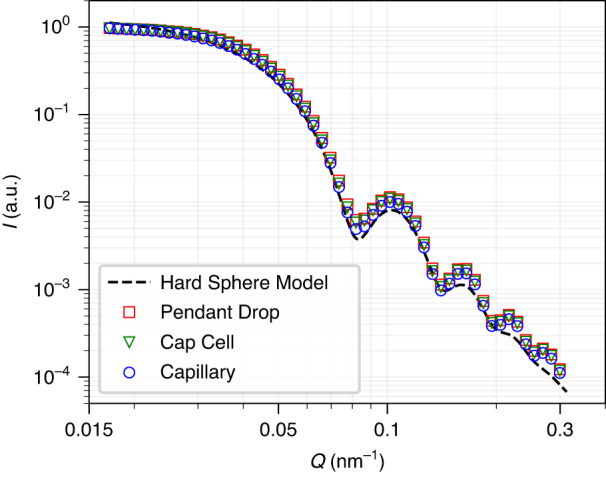
Comparison of SAXS results from the pendant drop, capillary, and cap cell. The scattering intensities are normalized to highlight the consistency among the intensity profiles. The dashed black line shows the calculated form factor from spherical nanoparticles with a Gaussian size distribution. The mean and standard deviation of the Gaussian distribution are 56.3 and 4.8 nm, respectively

Figure 3 compares SAXS results from the pendant drop setup in Fig. 1 with the capillary and Cap Cell setup. The SAXS results are extracted from a single SA-XPCS measurement with the Rigaku XSPA-500k detector, which consists of 100,000 frames of 1024 × 512-pixel images collected continuously at a 52 kHz frame rate. The 2D SAXS pattern was obtained from the time average of the 100,000 frames, which is equivalent to a single 1.92 s SAXS measurement performed on a rotating-anode x-ray source with identical x-ray flux. A 3 mm-diameter tungsten cylinder is placed immediately in front of the detector as a beam stop to prevent the transmitted X-ray beam from damaging the detector. The 1D SAXS intensity profile, i.e., scattering intensity as a function of momentum transfer Q, as shown in Fig. 3, was obtained from the azimuthal averages of the 2D SAXS pattern. At a fixed detector distance of 7.8 m, the minimum and maximum of Q is set by the size of the beam stop and the detector, respectively. Details regarding the XSPA-500k detector and the SA-XPCS analysis can be found in the Method and Materials section as well as in previous studies^31^. To facilitate the comparison, the scattering profiles in Fig. 3 are normalized by the maximum intensity within each measurement to account for the lack of precise control of sample volume, e.g., variation in the diameter of the quartz capillary and droplet size. Very good agreement can be seen in the 1D SAXS intensity profile among all three sample setups. For completeness, we have included the unnormalized 1D SAXS intensity profiles in Fig. S5a. We also note that the sample remains stable throughout the duration of the x-ray beam exposure, as indicated by the consistency among 1D SAXS profiles extracted from different time sections of the measurement in Fig. S5b.

**Fig. 4:**
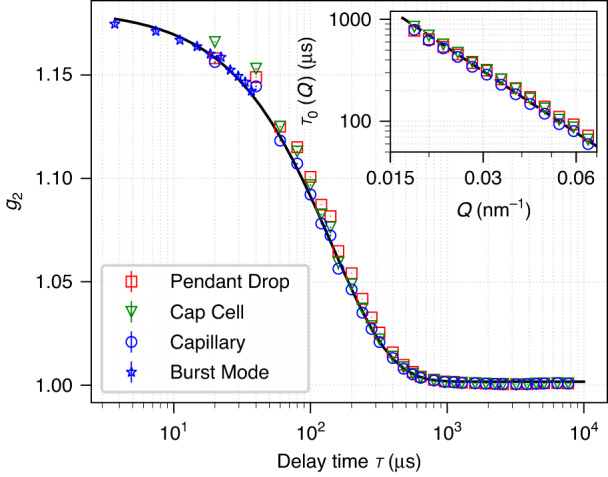
Intensity autocorrelation function g_2_ measured at Q = 0.028 nm^−1^ from all three sample setups. The black solid line shows the fit to the exponential function and the black dashed line in the inset figure shows the fit to the Einstein–Stokes equation. The 3.7-μs-resolved Burst Mode acquisition from the capillary setup is synchronized with the APS clock frequency to ensure that each detector frame has the same number of x-ray pulses

Figure 4 compares the XPCS intensity correlation function ${g}_{2}(\tau ,Q)$ at Q = 0.028 nm^−1^ from the pendant drop with the results from the capillary and Cap Cell setup. Both the XPCS and SAXS results are extracted from the same 1.92 s SA-XPCS measurement with 100,000 detector frames. While SAXS focuses on the overall structure information derived from the time-averaged scattering intensity, XPCS focuses on the fluctuation dynamics of said structure derived from the intensity correlation in time. Similar to DLS, the optical interference from coherent X-ray beams scattered by each nanoparticle results in a time-varying, “speckled” pattern on the detector, and the decay of intensity correlation ${g}_{2}$ of speckles as a function of frame spacing τ provides a quantitative probe of the time scale of nanoparticle dynamics. An advantage of XPCS compared to DLS is the use of a large pixelated area detector, where dynamics at different length scales can be selectively probed by evaluating ${g}_{2}$ at different detector regions. For example, ${g}_{2}\left(\tau ,Q\right)$ evaluated at the pixel with momentum transfer $Q$ corresponds to dynamics at a length scale of $2\pi /Q$. Details regarding the method, algorithm and implementation of SA-XPCS can be found in the Method and Materials section as well as Supplemental Materials. The inset of Fig. 4 shows the time constant ${\tau }_{0}(Q)$ extracted from the fit of ${g}_{2}=\beta\, {\rm{exp }}[-2\tau /{\tau }_{0}(Q)]+1$, where $\beta$ indicates the speckle contrast, a beamline-specific parameter^37^ that remains constant throughout the experiment. The good agreement of ${g}_{2}$ among all three sample setups across the entire measurable $Q$ range indicates that the pendant drop setup yields XPCS results consistent with the reference sample setup (sealed capillaries and Cap Cells). This combined with the consistency of the SAXS results in Fig. 3, successfully validated the use of pendant drop setup for SA-XPCS characterization of structure and dynamics in complex fluids.

Figure 4 also shows the “stitching” of ${g}_{2}$ between the Burst Mode (blue asterisks) and the Continuous Mode (blue circles) measured from the capillary setup, which extends the XPCS time resolution from 19.2 to 3.7 μs. When operating under the Burst Mode, the detector acquires a burst of 12 frames continuously at a minimum exposure time of 1 μs, and each burst of 12 frames is separated by a minimum readout time of 1 ms. In the previous work^36^, the burst frame acquisition was not synchronized with the APS clock frequency and the number of synchrotron x-ray pulses seen by each frame oscillated by 1, leading to the so-called “walking over the bunch” artifact. As a result, the use of Burst Mode is limited to APS bunch structures where the x-ray pulse spacing is much smaller than the exposure time per frame, so that this artifact from asynchronization can be safely neglected. In the current work, the start of each burst acquisition is externally triggered by the 272 kHz APS clock signal, leading to a 3.7 μs exposure time per frame. Since the 3.7 μs period is the time it takes for a single electron bunch to travel the entire storage ring (The 1104 m APS circumference divided by the speed of light), synchronizing the burst acquisition with the APS period allows Burst Mode to operate under any arbitrary x-ray bunch structures after the APS Upgrade (APS-U).

We note that burst mode is currently not compatible with pendant drop due to solvent evaporation during prolonged measurements. With the ~1% duty cycle and 20 times reduction in frame exposure time compared to the 52 kHz Continuous mode with 100% duty cycle, ${g}_{2}$ from burst mode requires 400 times more repeating measurements to accumulate the same statistics as Continuous Mode, which leads to a factor of 2000 increase in total measurement time. However, with the 100 times increase of coherent x-ray flux after APS-U, the number of repeating measurements for accumulating statistics will reduce by 10^4^. As a result, even a single measurement of Burst Mode after APS-U will achieve even better signal-to-noise ratio than 52 kHz Continuous Mode measurement at APS today.

**Fig. 5:**
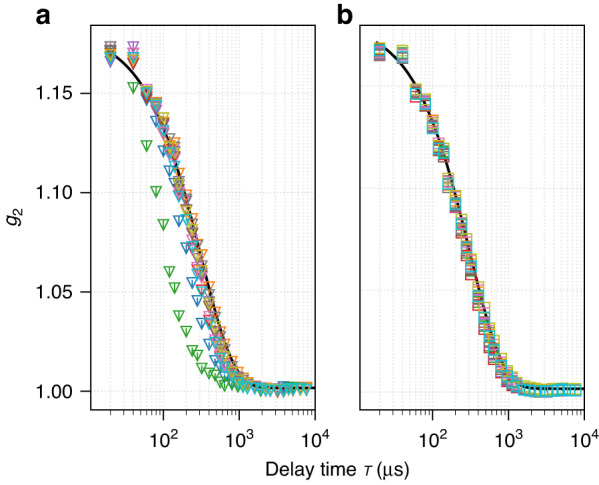
Stabilization of nanoparticle Brownian dynamics via suppression of solvent evaporation. Comparison of XPCS results from **a** pendant drop exposed to air and **b** pendant drop shielded by the cylindrical polycarbonate sleeve shown in Fig. 1. The same exponential fit in Fig. 4 is plotted in both (**a**, **b**) to highlight the effect of the polycarbonate enclosure

We also note that all SA-XPCS measurements in Figs. 2–4 were performed with the cylindrical polycarbonate sleeve surrounding the pendant drop, as shown in Fig. 1. For comparison, Fig. 5 shows ${g}_{2}$ from repeating measurements on the pendant drop setup with and without the polycarbonate sleeve. The beam position was fixed during the repeating measurements for both conditions. When the pendant drop is fully exposed, significant variation of ${g}_{2}$ is seen among repeating measurements, leading to large uncertainty in the XPCS results. A possible explanation is the evaporation-induced Marangoni convection^38^, which leads to additional fluid dynamics on top of the Brownian diffusion of silica nanoparticles. A previous study has shown that introducing additional velocity (e.g., controlled sample translation) can shift the ${g}_{2}$ falling edge if the velocity is commensurate with the intrinsic sample dynamics^39^. Therefore, a fully enclosed chamber filled with saturated solvent vapor is required for the implementation of a pendant drop setup at XPCS beamlines. Future implementation will also include gas inlets/outlets to enable vapor pressure control, which can be used to either suppress solvent evaporation for prolonged pendant drop measurements, or accelerate solvent evaporation for studies such as evaporation-induced crystallization^40^ and self-assembly^41,42^.

### Robotic automation of pendant drop setup

**Fig. 6:**
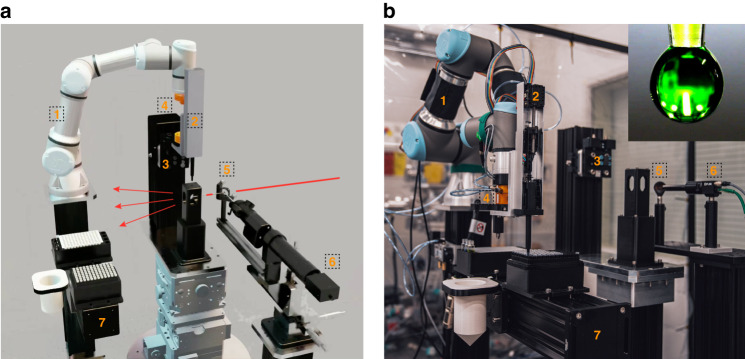
“Digital Twin” of the robotic pendant drop. **a** Snapshot of the robotic pendant drop in NVIDIA Isaac Sim. The electronic pipette is docked onto the mounting piece for the SA-XPCS measurement. The red arrows indicate the X-ray beams and the gray lines indicate the optical axes of the inline optical microscope. The key items (labeled 1–7) are explained in the Result section, and the technical details can be found in the Materials and Methods section. **b** Snapshot of the robotic pendant drop in the chemistry laboratory adjacent to beamline 8-ID-I. The electronic pipette is seen picking up a new pipette tip for liquid handling while attached to the end of the robotic arm via the tool changers. The inset figure shows an optical image of the pendant drop captured by the inline optical camera. Full motion of the robotic pendant drop can be found in Videos S1 and S2

A unique capacity of the pendant drop setup compared with liquid containers such as sealed capillaries is that the drop can be aspirated back into the pipette tip and disposed together with the tip at the end of the measurement, so that sample exchange does not require human intervention. In addition, the electronic pipette can be mounted on a robotic arm for automated liquid handling and sample preparation, which enables end-to-end, AI-executable SA-XPCS experimental workflows on complex fluids. Figure 6a, b present a snapshot of the operation of the robotic pendant drop setup in virtual reality (NVIDIA Isaac Sim) and in physical reality. A lightweight, small-footprint robotic arm (Item 1) was used to accommodate the tight working space at synchrotron x-ray beamlines. An electronic pipette (Item 2) generates a ~2 mm-diameter drop of liquid sample that hangs from the pipette tip. The pipette is detached from the robotic arm and docked on a dowel-pin mounting piece (Item 3) prior to the drop generation to eliminate μm-scale vibration from the robot joints that can contaminate XPCS results acquired from the μm-sized focused x-ray beam. The design also includes a 3D-printed chamber that encloses the pendant drop, as suggested by the observations from Fig. 5. The pick-up and docking action are performed using a compressed air-driven tool changer pair (Item 4). Engage/disengage actions between the pairing tool changer pieces is regulated via a five-way solenoid valve. To monitor the optical appearance of the drop during the alignment and SA-XPCS measurement, A 45^o^ reflective mirror with a 1-mm-diameter through-hole (Item 5) is placed upstream of the sample, and the reflection of the pendant drop is viewed with an optical microscope (Item 6). The through-hole on the mirror allows the X-ray beam to pass through, so that the optical image of the pendant drop can be captured simultaneously with the x-ray measurements. The sample preparation station (Item 7) includes a block that holds a 96-well PCR plate and multiple 3 mL vials (closest to the robot), a trash bin for used pipette tip, and a block that holds unused pipette tips (farthest from the robot). Technical details regarding the control workflow and the part number in the robotic pendant drop design can be found in the Method and Materials section.

The robotic pendant drop setup was demonstrated without an x-ray beam in the chemistry lab adjacent to Beamline 8-ID-I due to procedural and logistic challenges involving robotic work inside beamlines. Figure 6b shows the actual setup based on the simulation in Fig. 6a. The sample stage stack was replaced with an optical post of similar height, and the optical microscope was replaced with a video lens for higher refreshing rates. Video S1 shows a side-by-side comparison of the key steps between the simulation and the robotic motion in the chemistry lab. With the electronic pipette mounted on the robotic arm, the robotic pendant drop is able to mix two stock solution, extract the mixed sample, and dispense the drop for X-ray measurement. After a 3 s pause mimicking the x-ray measurement, the pendant drop is aspirated back into the pipette tip, and both the used sample and pipette tip were disposed. The full version of the robotic pendant drop motion in the chemistry lab can be found in Video S2.

We note that the work on the robotic pendant drop in this manuscript is performed entirely using open-source software. The UR3e robot arm is controlled using the urx Python library provided by the vendor (Universal Robot). The beamline electronics are controlled using the APS standard EPICS (Experimental Physics and Industrial Control System), and the built-in Python library pyEpics allows EPICS to coordinate with urx. The experimental orchestration was facilitated using the Workflow Execution Interface (WEI)^43^, a development of Argonne National Laboratory. WEI, a Python-enabled utility, is designed to bolster the automation and supervision of scientific activities. This tool utilizes a range of executors, one of which is the Robotic Operational System (ROS)^44^, to enhance inter-module communication. Notably, the WEI tool is adept at handling intricate workflows in both scientific research and laboratory settings. WEI allows for workflows to be specified using the YAML format. Each workflow is a sequence of actions or commands, executed by a distinct component, such as the UR3e or the beamline in our case. This modular approach provides a flexible platform that supports the design and execution of diverse workflows across different domains. In addition, we have integrated NVIDIA Isaac Sim for the simulation of robotic motion, leveraging its advanced physics simulation capabilities, artificial intelligence frameworks, and powerful graphics features. This allows us to leverage the established Python scientific ecosystem at the APS 8-ID-I beamline, paving the way to the collaborative development of autonomous AI protocols across multiple scientific user facilities. After the data acquisition is complete, the SA-XPCS analysis is performed automatically using the Python-based Data Management workflow^45^ developed at APS, and the data interpretation, including the rendering of Figs. 2–5 is performed using the Python-based pyXpcsViewer^46^. All Python scripts used in this work are uploaded to GitHub and the link is included in Supplemental Materials.

## Discussion

Robotic pendant drop provides a closed-loop, fully-automated experimental protocol for studying complex fluids with light scattering techniques. Sample preparation and characterization is highly efficient and repeatable, and the radiated aliquot is physically isolated from the reservoir to ensure zero cross-contamination from radiation damage. In addition, since the pendant drop is detached from the robotic arm throughout the XPCS measurement and is stable during alignment, as shown in Fig. 2, the sample can be translated continuously with the sample stage during the X-ray exposure, which will distribute the radiation dose over an area much larger than the beam size and greatly reduce the radiation-induced sample damage. The effect of stage translation on XPCS results can be easily corrected due to the uniform velocity profile^39^, in contrast to microfluidics, where the flow velocity shows large spatial inhomogeneity towards the wall of the microfluidic tube^47^. With a compact hardware design and open-source software platform, robotic pendant drop can be implemented not only in large user facilities such as synchrotron x-ray beamlines and free-electron lasers, but also at other laboratory-scale instruments such as rotating-anode SAXS and even tabletop instruments such as DLS. In addition, data from multimodal measurements can be analyzed in real-time, thanks to the development of open-source tools compatible with supercomputing facilities. With both open-source software and end-to-end automation, the AI supervising the robotic pendant drop will not only be able to ingest large-scale experimental results to advise on experimental plans, but also directly execute the experimental plans without human intervention, and acquire data that can be used to come up with the next experimental plans, therefore completing the cycle of “Self-driving lab”^48^ for autonomous design of soft materials with tailored properties.

The limitation of robotic pendant drop, similar to other containerless fluid setups, is that it does not provide temperature control with the same precision and ramping rate compared to metallic liquid containers (e.g., Cap Cells). In addition, the pendant drop needs to be surrounded by a saturated solvent vapor in enclosed chambers to suppress evaporation-induced Marangoni convection, which requires additional gas inlet/outlet implementation for vapor pressure control. Finally, the pendant drop is incompatible with samples that cannot be pipetted, e.g., solid and high-viscosity liquid. These technical limitations can be removed with the use of Cap Cells, which can also be assembled with robots, as demonstrated in Video S3. Automated assembly and exchange of Cap Cells will not only supplement the pendant drop capacity, but also facilitate mail-in operation at other high-throughput facilities such as SAXS and X-ray Diffraction beamlines. Future development will also leverage the unique in situ vapor pressure control capacity of the enclosed pendant drop setup to access non-equilibrium dynamics at liquid-liquid^49^ or liquid-solid interfaces^50^, where the small scattering volume of material interfaces can benefit from both the high spatial selectivity from the sub-μm focused x-ray beam and the drastic increase of coherent x-ray flux at next-generation synchrotron x-ray sources.

## Materials and methods

### Reference sample environments

The containerless pendant drop setup is validated by comparing the SA-XPCS results with reference liquid sample containers, i.e., sealed capillary and Cap Cell. Both containers were loaded into a 3 × 3 multi-sample temperature control unit from Quantum Northwest (Fig. S1), where the temperatures were set to match the room temperature at which the pendant drop measurements were performed. The thin-walled quartz capillaries were customized by Charles-Supper to have a shortened total length of 40 mm in order to accommodate the height limit of the Quantum Northwest setup (Fig. S2a). The liquid cell (Cap Cell) consists of three CNC-machined parts: an aluminum body and two identical polycarbonate caps. The aluminum body has tapped holes in the front and back, and the internal threads on the tapped holes match the external threads on the polycarbonate caps. The polycarbonate caps follow the same design as generic hollow set screws (McMaster-Carr, Part Number 91318a712), except that one side is covered with a window. The polycarbonate cap is machined as a single piece and the window thickness is 0.13 mm after vapor polishing, which is performed post-fabrication to both improve optical clarity and reduce SAXS scattering background (Fig. S2b). During the sample loading, one of the polycarbonate caps is threaded onto the aluminum body to form a small basin at the center, where the sample is either pipetted into the basin, or scooped if the sample is too viscous/solid to be pipetted. Tests have shown that water sealed in the cell does not exhibit significant leakage for up to 40 h at ambient environment (Fig. S3), and up to 4 weeks if the seal is reinforced on the exterior with transparent adhesive films (Fig. S4). Compared to capillaries, the Cap Cells are more compatible with solid or gel samples^51^, and the metal body allows for a much faster temperature ramping rate and shorter thermal equilibration time^52^, both of which are essential for the study of non-equilibrium, phase-transitioning structural dynamics in complex fluids.

### Rigaku XSPA-500k detector

The transmitted x-ray scattering intensities were collected 7.8 m downstream from the sample using Rigaku XSPA-500k, a 1024 × 512 pixelated single-photon-counting detector with 76-μm pixel size^53^. XSPA has three acquisition modes: Long Mode, Continuous Mode, and Burst Mode. The Long Mode has a maximum frame rate of 8.5 kHz with no upper limit on the total number of frames; The Continuous Mode has a maximum frame rate of 52 kHz and a maximum frame number of 100,000; The Burst Mode has a maximum frame rate of 1 MHz and a maximum frame number of 120,000, i.e., each burst has 12 continuous frames with a minimum exposure time of 1 μs per frame, and each burst is separated by a minimum readout time of 1 ms, leading to a total measurement time of 12–15 s with 1–5% duty cycle. Combining the three acquisition modes leads to gapless coverage of time scales from μs to tens of seconds^36^ and potentially up to thousands of seconds if needed.

### SA-XPCS measurement and analysis

In an SA-XPCS measurement, an optically-coherent X-ray beam generated by an undulator X-ray source at a synchrotron or a free-electron laser is focused on a ~μm-sized spot on the sample. The scattered X-ray beam is collected ~10 m downstream of the sample with a pixelated detector, while the directly transmitted X-ray beam is blocked using a mm-diameter thick metal cylinder (e.g., beam stop) to protect the detector. The sample-to-detector distance is determined so that the optical interference pattern is sufficiently sampled by the pixelated detector, i.e., the Fraunhofer diffraction angle from the focused x-ray beam (x-ray wavelength divided by beam size) matches the solid angle spanned by the detector pixel (pixel size divided by sample-to-detector distance). During the SA-XPCS measurement, the detector operates like a video camera and the x-ray photons arriving at the detector are registered pixel-by-pixel and frame-by-frame, leading to a movie of “flickering speckles”. For the current work, each SA-XPCS measurement consists of a movie of 100,000 frames, each with 1024 × 512 pixels and 19.2-μs exposure time, collected continuously over the course of 1.92 s at a frame rate of 52 kHz.

Both SAXS and XPCS analysis are performed on the same SA-XPCS data set. The SAXS analysis is the same as SAXS performed on a rotating anode (a.k.a. lab x-ray source) or a synchrotron bending-magnet x-ray source that does not have sufficient optical coherence for XPCS measurement. Here, the 100,000 detector frames are averaged in time to produce the equivalent of a single 1.92 s exposure. This 2D detector image is then averaged azimuthally over a Region of Interest on the detector (i.e., a ring of pixels centered around the direct beam position, also referred to as ROI) with a width of approximately one pixel to produce the 1D SAXS scattering intensity shown in Fig. 3. This one-pixel-wide, fine-grained ROI is also known as the “static ROI” and all pixels within the ROI is considered to have the same Q. Note that in Fig. 3, only one per five data points is plotted to improve the clarity of the figure as well as facilitate comparison among different sample conditions. More details can be found in the GitHub repository: Data_Analysis/Figure_scripts/Plot_SAXS.ipynb,

The XPCS analysis is performed in the following steps: First, G2, IP, and IF are calculated at each pixel using the same method as in single-pixel DLS. Numerical details regarding the G2, IP, IF calculation, and the associated multitau frame binning are well-documented and lengthy, so we refer the readers to DLS literatures^54^ and the Supplemental Materials of our previous work^31^. The G2, IP, and IF are then averaged within the same static ROIs in the previous SAXS analysis. The ratio of the pixel-averaged G2, IP, and IF yields the intensity autocorrelation function g_2_ at the Q of the static ROI, i.e., ${g}_{2}={\left\langle G2\right\rangle }_{Q}/\left({\left\langle {IP}\right\rangle }_{Q}\cdot {\left\langle {IF}\right\rangle }_{Q}\right)$. This g_2_ is then binned over approximately every ten static ROIs (also called the dynamic ROI) to further improve the statistics. The error bar of g_2_, on the other hand, is evaluated using the standard deviation of the unaveraged, pixel-wise G2/IP/IF within the dynamic ROI.

A Python package including the function library and test data for single-CPU XPCS analysis, can be found in the Cloud database provided in the Supplementary Information. Although the performance is ~100 times slower compared to the production-level GPU code currently deployed at APS 8-ID Beamline, the algorithm is identical, and the result file can also be opened and visualized using the pyXpcsViewer^46^.

### Robotic pendant drop

The robotic pendant drop setup provides a fully-automated solution for liquid sample preparation, alignment, exchange, and disposal at a synchrotron x-ray beamline. Simulation of the robotic pendant drop is performed using NVIDIA Isaac Sim, a robotics software platform developed by NVIDIA that offers a comprehensive set of tools for simulation, training, and deployment of autonomous systems and robotic applications. One of the standout features of the NVIDIA Isaac Sim is its advanced physics simulation capabilities, which allow for realistic and accurate simulation of robot behavior. Additionally, NVIDIA Isaac Sim incorporates a powerful artificial intelligence framework that allows robots to perceive, reason, and act intelligently in real-world environments. This framework allows developers to train robots using various advanced AI techniques, such as reinforcement learning and deep learning, enabling them to perform complex tasks and operate safely in unstructured environments. NVIDIA Isaac Sim also boasts powerful graphics features, leveraging high-performance GPUs to enable real-time rendering of complex virtual environments with high-fidelity visuals. These graphics features make NVIDIA Isaac Sim an ideal platform for creating visually compelling and accurate simulations of robotic systems, which is particularly important for training robots to operate in complex environments.

Technical parameters of all items in Fig. 6a are listed below:

1. UR3e robotic arm from Universal Robot;
2. P300 GEN2 electronic pipette from Opentrons. The plunger inside the electronic pipette is driven by a bipolar motor driver, ACS StepPak SPD32M, which communicates with the beamline via EPICS;
3. Mounting piece with dowel pins, 9120-TSS-HBQ-7123 and 9120-TSS-MMB-7124 from ATI;
4. QC-11 compressed air-driven tool changers from ATI. The compressed air is regulated using a five-way solenoid valve (V60P517A-A213JB from Norgren). The valve is controlled using a LabJack T7 module which communicates with the beamline via EPICS;
5. 25.4-mm-diameter, aluminum-coated 45^o^ reflective mirror with 1 mm-diameter through-hole from OptoSigma;
6. 12.5X High Precision Zoom Lenses from Edmund Optics. Optical images from the microscope is captured by a Blackfly S CCD camera from Teledyne FLIR, which communicates with the beamline via the EPICS plugin of ImageJ. For the robotic integration in Fig. 6b, Item (5) is replaced with a video lens, i.e., InfiniStix 1.0X with a 94 mm working distance from Edmund Optics;
7. Sample preparation station. From the robot pedestal to the end of the optical post: 96-well block with 3 ml vial holders, trash bin for used pipette tips, and fresh pipette tips holder.

All three items in (7) and the droplet enclosure were 3D-printed for rapid prototyping. The UR3e robot is interfaced via the urx Python library provided by Universal Robot, and all beamline components communicating with EPICS is interfaced via the pyEpics Python library. The urx library is compatible with the pyEpics library, and the entire robotic pendant drop is programmed and operated 100% using Python and within a single conda environment. The Python script for the control of the robotic pendant drop is posted on GitHub (link in the Supplemental Materials).

### Supplementary information

Video S1

Video S2

Video S3

Supplementary Materials for “Robotic Pendant Drop: Containerless Liquid for μs-resolved, AI-executable XPCS”
