# Supplementary material for: Robotic pendant drop: containerless liquid for μs-resolved, AI-executable XPCS
Source: Light Sci Appl. 2023 Aug 18;12:196. doi: 10.1038/s41377-023-01233-z (PMC10439219; doi:10.1038/s41377-023-01233-z)
Supplement: Supplementary file 4 — Supplementary Materials for “Robotic Pendant Drop: Containerless Liquid for μs-resolved, AI-executable XPCS” [file 41377_2023_1233_MOESM4_ESM.pdf]

Supplementary Materials for "Robotic Pendant Drop: Containerless Liquid for µs-resolved, AI-executable XPCS"

Cloud data base:

https://anl.box.com/s/kp7t46522r0sli7gy520n7o4295adev5

Auxiliary materials and data for the manuscript, including:

1. `Video_Demo`:
   Video S1~S3 in high resolution.
2. `Isaac_programs`:
   All files used in the Nvidia Isaac Sim.
3. `XPCS_Analysis_Example`:
   Python scripts for single-CPU XPCS analysis. An example Rigaku XSPA-500k detector data set and the metadata files are included. The algorithm is identical to the GPU-cluster XPCS code used at the APS 8-ID-I beamline.

GitHub repositories:

https://github.com/qzhang234/PubCode-2022-Pendant_Drop

This repository houses that SA-XPCS data and analysis code, including:

1. XPCS result files from APS 8-ID-I;
2. Python scripts for importing the results and rendering the figures in the manuscript;
3. **Figure 2~5** in vector graphics.

https://github.com/AD-SDL/rpl_wei/tree/main

This repository houses the workcell execution interface at the Rapid Prototyping Lab (RPL) of Argonne National Laboratory (https://rpl-wei.readthedocs.io/en/latest/). It includes the scripts that send the ROS action messages to start the WEI server and parse the workflow files to be executed on the UR robot. WEI is a Python-based tool for automating and managing sequences of instrument and computation actions (workflows) in a modular environment in which a variety of hardware and software components implement common interfaces. The repository includes:

1. `rpl_wei/rpl_wei/core/`
   Contains the core components of the WEI code base.
2. `rpl_wei/scripts/run_wei_server.sh`
   Starts the WEI server.

https://github.com/AD-SDL/ur_module/tree/main

This repository houses the ROS node and driver package for UR robot, including:

1. `ur_driver`
   Python scripts for driving the UR robot, tool changer, camera and the pipette built on the `urx` and `pyepics` library.
2. `ur_client`
   A ROS node that constantly keeps track of the robot state and listens for action messages from WEI executers. When the action message is received by the ROS node, it is immediately executed on the robot using `ur_driver`.

https://github.com/AD-SDL/8IDI_workcell/tree/main
This repository houses the files pertaining to the "workcell" and "workflow" components of the droplet experiment, along with the Python script responsible for initiating the workflow execution. The workcell, specified as a YAML file, delineates the constituent modules comprising the workcell and the corresponding static infrastructure to be utilized by the workflow. The workflow, also represented as a YAML file, outlines a predefined sequence of actions to be executed on one or more modules within the workcell. The repository includes:

1. `droplet_workcell/workflows/droplet_exp.yaml`
   Steps of the experiment to be executed by WEI, written in human readable format.
2. `droplet_workcell/run_droplet.py`
   Starts the experiment by loading the workflow file to WEI executer.

Figures:

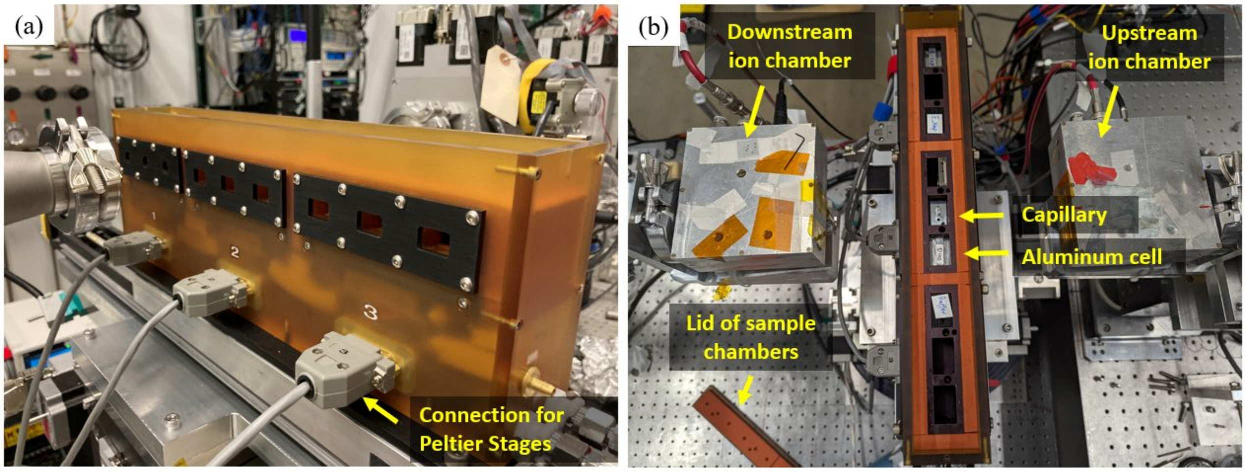

Figure S1 (a) Front view (viewed from the downstream direction of the x-ray beam) of the Quantum Northwest setup (QNW 3×3) at APS Beamline 8-ID-I. The setup holds a total of 9 cells. Every 3 cells share the same temperature zone. Each temperature zone is controlled using a Peltier stage and a resistive heater. **(b)** Top view of the QNW setup. The cell slots are 35.6 mm (height) × 25.4 mm (width) × 15.7 mm (thickness in the x-ray direction). The anodized aluminum holder (black grid) in each temperature zone has 3 cell slots. The spacing between the anodized aluminum holders is padded with thermally insulating materials. Two ion chambers are placed upstream and downstream of the QNW setup to monitor the x-ray flux and sample transmission coefficient simultaneously during the SA-XPCS measurements.

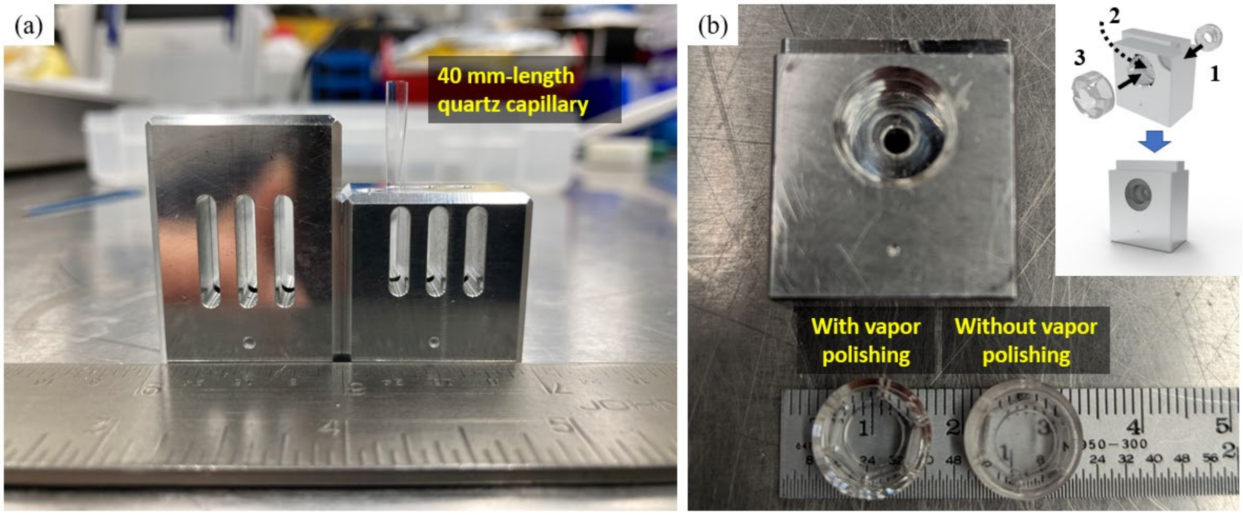

Figure S2 (a) The capillary setup. The customized thin-walled quartz capillary from Charles-Supper has a reduced total length of 40 mm including the funnel end to accommodate for the height of the QNW setup. The capillary can slide into the aluminum blocks, and the aluminum blocks can be inserted into slots in the QNW as shown in **Figure S1b**. The taller aluminum block on the left is designed for 40 mm-long capillaries without the loading funnel end. The dents on the bottom of both blocks match the bumps inside the QNW holder to lock the blocks in place during stage translation. **(b)** The Cap Cell setup. The sample reservoir has a diameter of 3 mm. The thickness of the reservoir along the beam direction can be customized from 0.5 mm to 4 mm to match the x-ray attenuation length at different photon energies. The threads of the tapped hole on the aluminum holder match the CNC-machined polycarbonate cap. The optical clarity of the polycarbonate cap can be improved via chemical vapor polishing. The Cap Cell assembly is illustrated in the inset figure: 1) Screw one cap into the aluminum block; 2) Pipette the sample into the reservoir if the sample is liquid, or scoop out the sample and fill the reservoir if the sample is highly viscous or solid; 3) Screw the other cap into the aluminum block to form a seal. Spacer window material (e.g. Kapton films) can be inserted between the sample and the polycarbonate caps if sample is not compatible with polycarbonate.

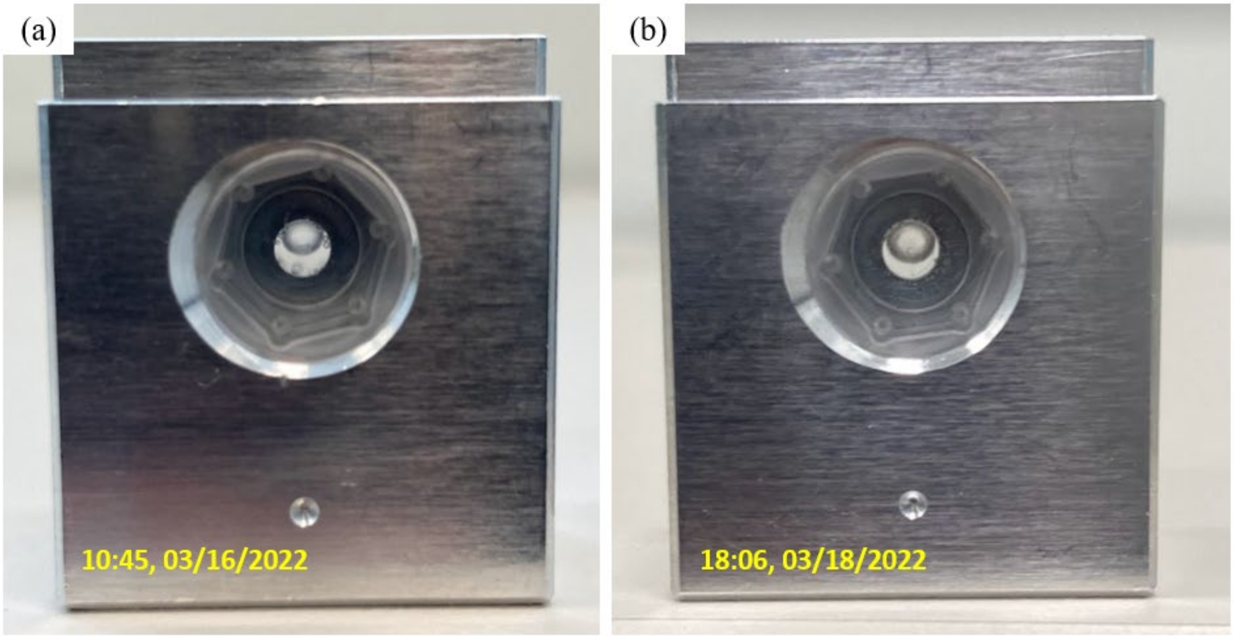

Figure S3 Leak test of the Cap Cell setup. The Cap Cell can hold water for up to 40 hours in the ambient environment without significant leakage.

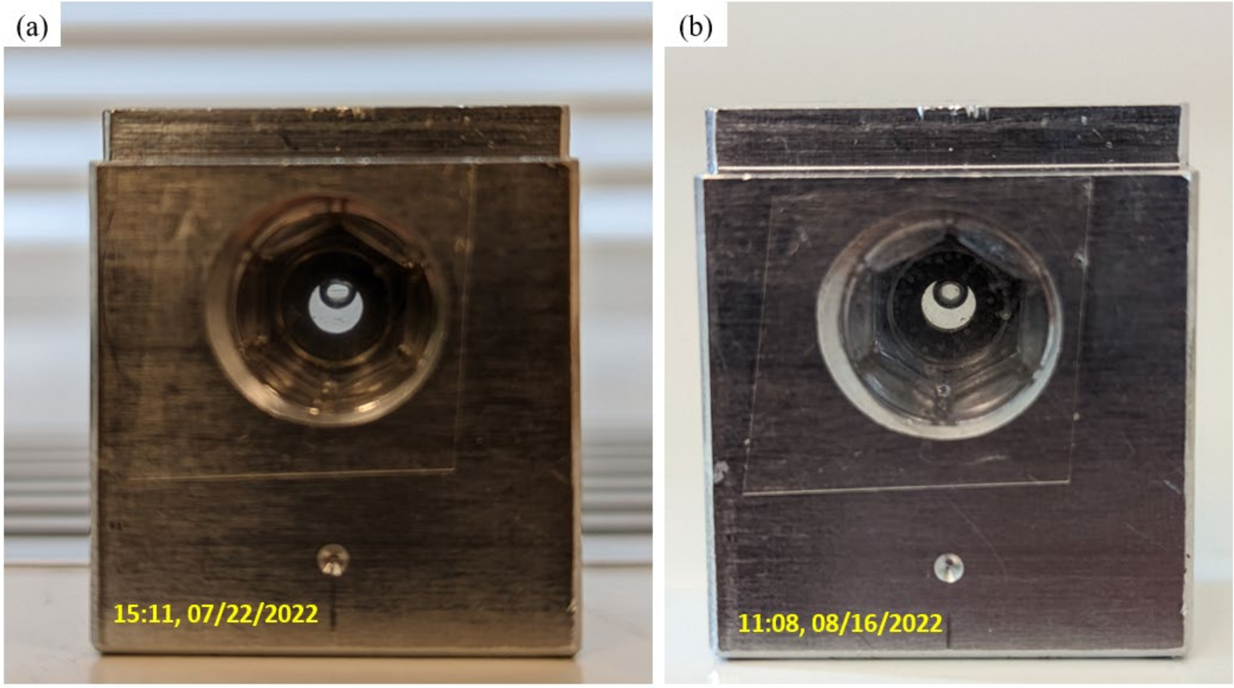

Figure S4 Leak test of the reinforced Cap Cell setup. The Cap Cell can hold water in the ambient environment for as long as 4 weeks if the seal is reinforced with adhesives, e.g., VIEWSeal pressure-activated silicone/polyolefin sealing film from Greiner.

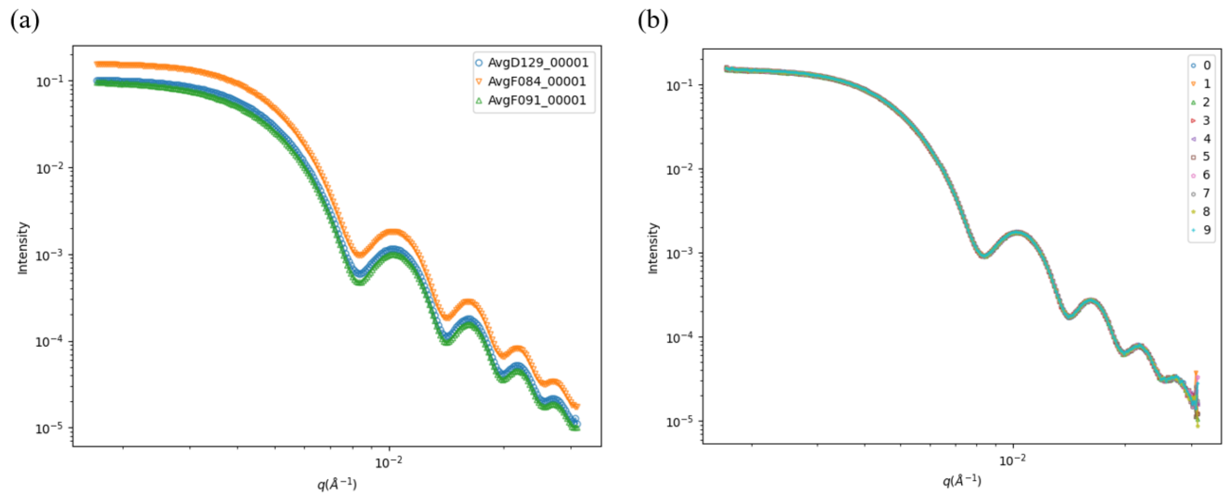

Figure S5 (a) Comparison of unnormalized 1D SAXS intensity profiles from the capillary (green upward triangles), Cap Cell (blue circles), and pendant drop (orange downward triangles). The unit in y axis is number of photons per pixel per detector frame. Offset in the scattering intensity arises from difference in the sample thickness, i.e. the volume of sample along the x-ray beam path. **(b)** "Stability Plot" from the pendant drop result. The 100,000 detector frames were sequentially divided into 10 sub-sections of 10,000 frames, and each curve (0-9) corresponds to the SAXS intensity profile from a single sub-section. Consistency in stability plot indicates that the sample remained stable throughout the 1.92 second of synchrotron x-ray beam exposure.

Note that both figures can be reproduced by loading the XPCS result files on the GitHub repo into pyXpcsViewer. The figure legend in **(a)** indicates the file name. The source code of pyXpcsViewer, including instructions on installation and use of the software, can be found at: https://github.com/AdvancedPhotonSource/pyXpcsViewer.
